# Physical and Developmental Characteristics of Elite Under-19 Female Cricketers: Positional Differences, Relative Age Effects, and Maturity

**DOI:** 10.3390/sports14080352

**Published:** 2026-08-17

**Authors:** Sibi Walter, Gregory King, Harrison Jones-Park, Dayle Shackel, Ben Ramsay, Elena Moltchanova

**Affiliations:** 1 Faculty of Health, University of Canterbury, Christchurch 8140, New Zealand; 2 Canterbury Cricket, Christchurch 8140, New Zealand; 3 New Zealand Cricket, Christchurch 8140, New Zealand; 4 Statistics Department, University of Canterbury, Christchurch 8140, New Zealand

**Keywords:** cricket, fitness testing, strength test, athletic performance

## Abstract

Background: Women’s cricket is rapidly expanding at the elite youth level, yet limited research has comprehensively examined the physical, positional, and developmental characteristics underpinning performance in elite under-19 (U19) female players. In particular, the role of biological maturation and relative age effects in shaping physical performance within this population remains poorly understood. Objective: This study aimed to characterise the anthropometric and physical profile of elite U19 female cricketers, examine positional differences, assess the presence of relative age effects (RAE), and evaluate the applicability of maturity offset estimation in this cohort. Methods: Ninety elite U19 female cricketers representing six regional high school teams were assessed using a cross-sectional design. Anthropometric measures, strength, power, sprint performance, and agility were evaluated. Maturity status was estimated using both the original and modified maturity offset equations, and birth quartiles were used to assess RAE via a chi-squared test. Between-group differences were analysed using ANOVA. Results: Statistically significant positional differences were observed for vertical jump, isometric strength, and sprint performance (*p* < 0.05), with spin bowlers demonstrating lower power and slower sprint times, and seam bowlers exhibiting greater maximal strength. No differences were observed in anthropometry or agility. Birth quartile distribution was relatively uniform, indicating no clear RAE. Maturity offset estimates indicated that players were well beyond peak height velocity; however, both original and modified estimates demonstrated limited variability and a tendency to overestimate maturity timing in this cohort. Conclusions: Elite U19 female cricketers demonstrate role-specific physical profiles driven primarily by strength, power, and speed rather than anthropometry or maturity status. The absence of a clear RAE and limited applicability of maturity offset methods highlight the need for performance-based, position-specific approaches to training and talent identification in female cricket.

## 1. Introduction

Women’s cricket has undergone rapid expansion in participation, visibility, and professionalisation over the past decade, driven by increased investment, structured development pathways, and high-profile international competitions [1]. The emergence of global tournaments such as the ICC Women’s Under-19 World Cup has intensified competition for selection into elite youth squads, thereby amplifying the importance of evidence-based approaches to talent identification, athlete development and injury prevention [2,3,4,5,6,7]. Within this context, a comprehensive understanding of the physical and developmental characteristics underpinning performance in elite adolescent female cricketers is essential.

Cricket is an intermittent, multifaceted sport characterised by prolonged periods of low-intensity activity interspersed with repeated high-intensity efforts such as sprinting, bowling, batting, and multidirectional fielding movements. These demands vary substantially according to match format and, critically, according to player role [8]. Seam bowlers are exposed to repeated high-intensity run-ups and ground reaction forces, necessitating high levels of strength and power [9,10]. In contrast, batters rely on repeated sprint ability, agility, and upper-body power to execute strokes and run between wickets [11]. Consequently, the physical characteristics required for optimal performance are both diverse and role-specific.

Despite recognition of these positional demands among men, the current scientific literature provides only a fragmented understanding of cricket-specific physical profiles among women [12]. This is problematic, as extrapolating findings from male athletes or single subgroups fails to account for sex-specific physiological characteristics and the unique developmental trajectories observed in adolescent female athletes [13]. Furthermore, the lack of role-specific data in female cricket restricts the ability of strength coaches to design targeted interventions that reflect the demands of playing positions [14].

In youth sport, physical performance is not solely determined by training exposure and technical skill but is also strongly influenced by biological maturation [15]. The adolescent period is marked by rapid changes in stature, body composition, and neuromuscular coordination, all of which contribute to the development of strength, power, and speed [16]. Athletes who mature earlier often demonstrate temporary physical advantages over their peers, including greater height, muscle mass, and force production capacity [15]. These advantages may translate into improved sport-specific performance characteristics, such as increased bowling velocity, enhanced sprint performance, and greater hitting distance in cricket. However, such benefits are frequently transient and may not reflect long-term athletic potential.

The influence of biological maturation on performance introduces important implications for talent identification and development systems [17]. Maturity-related differences may bias selection processes, whereby early-maturing athletes are preferentially selected due to their current physical superiority, while late-maturing athletes may be overlooked despite possessing comparable or greater long-term potential [18,19]. To account for these effects, non-invasive methods such as maturity offset estimation, which predicts years from peak height velocity (PHV), are commonly employed in youth sport research [20,21,22]. These methods offer practical alternatives to more invasive measures of maturation; however, their validity in specific populations, particularly older adolescent female athletes and elite cohorts remains uncertain. Reduced variability in maturation status within such groups may limit the sensitivity of maturity offset equations, raising questions regarding their applicability in high-performance settings [20,22,23].

In addition to biological maturation, another well-documented source of bias in youth sport is the relative age effect (RAE) [24]. RAE arise due to the structuring of youth sport into annual age-group categories, leading to systematic advantages for athletes born earlier in the selection year. These relatively older athletes are often more physically and cognitively developed than their younger peers within the same cohort, increasing their likelihood of selection into talent pathways [25]. RAE have been consistently observed across a range of sports, including cricket, and are particularly pronounced during adolescence [26]. In male cricket, evidence suggests that players born in the early months of the selection year are significantly overrepresented in youth talent pathways, benefiting from increased access to coaching, competition, and developmental resources [25].

However, the presence and magnitude of RAE in female cricket remains less clear. Limited investigations within female cricket have identified RAE at youth levels, yet comprehensive analyses across elite developmental cohorts remain scarce [24]. Furthermore, the interaction between RAE and player roles, physical characteristics, and maturation status has yet to be adequately explored in female cricket populations.

The rapid growth and restructuring of female cricket development pathways further underscores the importance of understanding these factors. Increasing opportunities for participation and professional progression have intensified the need for robust, evidence-based talent identification frameworks. However, current systems continue to rely heavily on subjective assessment and short-term performance indicators, which may be influenced by both relative age and maturation status. This raises concerns regarding the potential misidentification or exclusion of talented athletes who develop later or fall outside traditional selection biases.

Collectively, these gaps highlight the need for a comprehensive, integrated investigation of physical and developmental characteristics in elite adolescent female cricketers. Specifically, there is a lack of research examining how physical profiles vary across playing roles, how relative age distributions are represented within elite cohorts, and whether commonly used maturity offset equations are appropriate for this population. Addressing these areas is crucial for improving the accuracy of talent identification, optimising training prescription, and supporting the long-term development of female cricket players.

Therefore, the aims of this study were fourfold: (1) to characterise the anthropometric and physical profile of elite under-19 female cricketers; (2) to examine differences in physical characteristics across playing positions; (3) to determine the prevalence of relative age effects through analysis of birth quartiles; and (4) to evaluate the applicability of maturity offset estimation within this cohort. By integrating performance profiling with developmental considerations, this study seeks to provide novel insights that can inform evidence-based practice in the rapidly evolving landscape of women’s cricket.

## 2. Materials and Methods

### 2.1. Study Design

A cross-sectional observational design was employed to characterise the anthropometric, physical, and developmental profiles of elite under-19 (U19) female cricketers and to examine differences across playing positions, relative age distributions, and maturity status. Data collection took place during the national high school cricket tournament, which represents the highest level of competition for school-aged female cricketers in the country. All testing procedures were standardised and conducted during a single testing session following a structured injury-prevention warm-up and familiarisation protocol.

### 2.2. Participants

Participants were elite U19 female cricketers (*N* = 90) selected to represent one of six regional First XI high school teams. Selection into these teams was based on performance in inter-school and regional competitions, where top-performing athletes were identified through competitive match play and talent identification pathways. Inclusion criteria required participants to be actively competing in the national tournament and free from any injury or medical condition that could affect performance at the time of testing. Ethical approval was obtained from the National Cricket Board and the University of Canterbury Human Research Ethics Committee (HREC: 2022/107). Written informed consent was obtained from all participants as well as their parents or guardians.

### 2.3. Study Setting

Testing was conducted on level grass fields adjacent to the national cricket training centre under consistent environmental conditions. Participants wore standard cricket training attire and spiked footwear to ensure ecological validity and minimise disruption to typical movement patterns. All assessments were administered by trained researchers using standardised procedures to ensure consistency across participants.

### 2.4. Player Classification

Players were categorised into positional roles based on coach classification and definitions set by the national cricket association. Batters were defined as players whose primary role was batting and who were not regularly required to bowl or keep wickets. Bowlers were classified as players whose primary role was to deliver overs and were further grouped as pace or spin bowlers where applicable. All-rounders were defined as players who consistently contributed to both batting, typically within the top seven positions, and bowling, delivering at least 10% of their team’s overs. Wicketkeepers were identified as players performing specialised wicketkeeping duties, positioning themselves relative to the speed of the bowler.

### 2.5. Anthropometric Assessments

Anthropometric measurements were conducted using standardised protocols. Body mass was measured using an electronic scale (WS207PMSG, Wedderburn, Christchurch New Zealand) and standing and sitting heights were measured using a portable stadiometer (WMHM200P, Wedderburn, Christchurch, New Zealand). All measurements were taken with participants in minimal clothing and without footwear. Sitting height was assessed with participants seated upright on a bench, with hips and knees flexed at 90°, and feet placed flat on the ground. Leg length was calculated as the difference between standing height and sitting height [23].

### 2.6. Physical Performance Testing

#### 2.6.1. Lower-Body Power

Lower-body power was assessed using a vertical jump and standing broad jump. The jump was measured using a vertical jump yardstick (Swift Performance, Wacol, Australia), where participants performed a maximal jump and reached to displace the highest vane possible [27]. Horizontal power was assessed using the broad jump, in which participants jumped forward maximally from a standing position, with the distance measured from the starting line to the heel position at landing using a fibreglass measuring tape [28].

#### 2.6.2. Upper-Body Power

Upper-body power was evaluated using a seated medicine ball chest throw (SMBT) with a 2 kg ball (Hart, Christchurch, New Zealand). Participants sat with their back firmly against a wall and projected the ball forward, with the maximum distance from the wall to the initial landing point recorded [29].

### 2.7. Strength Assessments

Maximal strength was assessed using an isometric mid-thigh pull (IMTP) and grip strength measurements. The IMTP was performed using a back–leg–chest dynamometer (Saehan, Seoul, Republic of Korea), with participants instructed to exert maximal force in accordance with established testing guidelines [30]. Grip strength was measured using a handheld dynamometer (Jamar Smart, Warrenville, IL, USA), with participants performing three alternating maximal efforts for each hand to minimise fatigue. The highest value recorded for each hand was used for analysis [31].

### 2.8. Sprint Performance

Sprint performance was assessed over distances of 10 m, 20 m, and 30 m using electronic timing gates (Speedlight, Swift Performance, Wacol, Australia). Participants completed two maximal sprint trials on natural grass while wearing cricket spiked footwear, with the fastest trial retained for analysis.

### 2.9. Agility

Change-of-direction ability was assessed using the *T*-test, performed according to established protocols. Performance time was recorded via slow-motion video capture using an iPad Air 5 (Apple Inc., California, CA, USA), and the fastest trial was used for analysis [32].

### 2.10. Maturity Assessment

Biological maturity was estimated using both the original [21] and modified Moore [22] regression equations to calculate maturity offset, representing years from peak height velocity (PHV). The modified Mirwald equation for females was used as the primary method due to its suitability for older adolescent populations, while the original equation was used for comparison [20]. Age at PHV (APHV) was subsequently calculated as chronological age minus maturity offset. Based on APHV derived from the modified equation, participants were classified as early, on-time, or late maturers. Athletes reaching PHV before 11 years were classified as early maturers, those between 11 and 13 years as on-time maturers, and those above 13 years as late maturers [33].

### 2.11. Relative Age Effect

Relative age was determined using calendar-year birth quartiles (Q1 = January–March; Q4 = October–December), consistent with 1 January as the selection cut-off for the national secondary school cricket tournament. This approach also reflects the absence of a fixed school-entry cutoff in New Zealand [24,25,26].

### 2.12. Statistical Analysis

Pearson correlation coefficients were calculated to examine relationships among anthropometric characteristics, strength and power measures, sprint and agility performance, and maturity indicators. Differences in anthropometric and physical performance characteristics between playing positions were assessed using one-way analysis of variance (ANOVA), followed by Tukey’s post hoc comparisons where appropriate. Normality and homoscedasticity of residuals were assessed via diagnostic residual and QQ plots. To examine the presence of a relative age effect (RAE), a chi-squared goodness-of-fit test was used to compare the observed birth quartile distribution against an expected uniform distribution (25% per quartile). Differences in quartile distributions between playing positions were assessed using a chi-squared test of independence [24,25,26]. Statistical significance was set at *p* < 0.05. All analyses were conducted using R (version 4.4.0; R Foundation for Statistical Computing, Vienna, Austria).

## 3. Results

### 3.1. Participant Characteristics

A total of 90 elite under-19 female cricketers were assessed across five playing positions with a mean age of 16.3 ± 1.3 years. In the observed cohort, every all-rounder played a batter–seam bowler role and there was no spin bowler–batter combination. The position-specific and overall sample statistics—means and standard deviations for the continuous outcomes of interest and frequencies and percentages for categorical ones—are shown in Table 1. Across the entire sample, players demonstrated well-developed physical characteristics, with a mean height of 167.5 ± 6.3 cm, body mass of 65.5 ± 10.6 kg, vertical jump of 36.7 ± 5.8 cm, and IMTP strength of 95.5 ± 30.9 kg as shown in Table 1.

### 3.2. Relationships Between Physical Performance and Maturity

**Figure 1:**
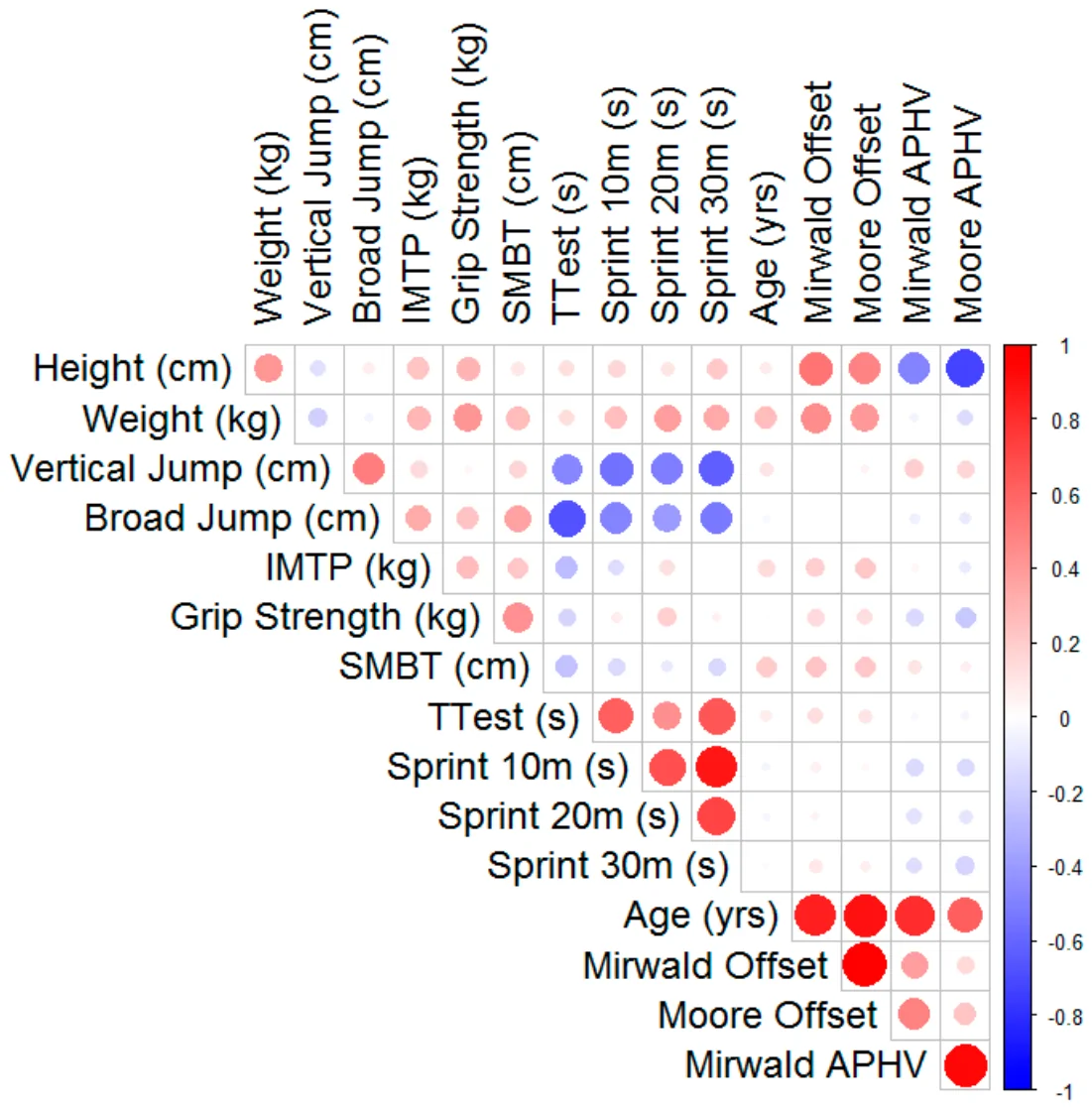
Pearson’s correlation matrix showing relationships between anthropometry, physical performance, and maturity. Notes: cm, centimetres; kg, kilograms; s, seconds; m, metres; yrs, years; APHV, Age at Peak Height Velocity; IMTP, isometric mid-thigh pull; SMBT, seated medicine ball throw.

Correlations between variables of interest are visualised in Figure 1. Lower-body power (vertical jump and broad jump) was moderately to strongly associated with sprint performance, with greater power linked to faster sprint times. Strength measures (IMTP and grip strength) were positively associated with anthropometric characteristics—particularly body mass. Maturity indicators were strongly related to age and body size but showed only weak associations with physical performance measures. This suggests that, within this cohort of elite U19 female cricketers, maturity status had a limited influence on performance outcomes.

The estimated effect sizes η2, test statistics and *p*-values for the group comparisons via ANOVA are listed in Table 2. Anthropometric characteristics were, on average, similar across positions, with no significant differences in stature observed (F (4,85) = 0.83, *p* = 0.510) as shown in Table 1 and Table 2.

### 3.3. Differences Between Playing Positions

**Figure 2:**
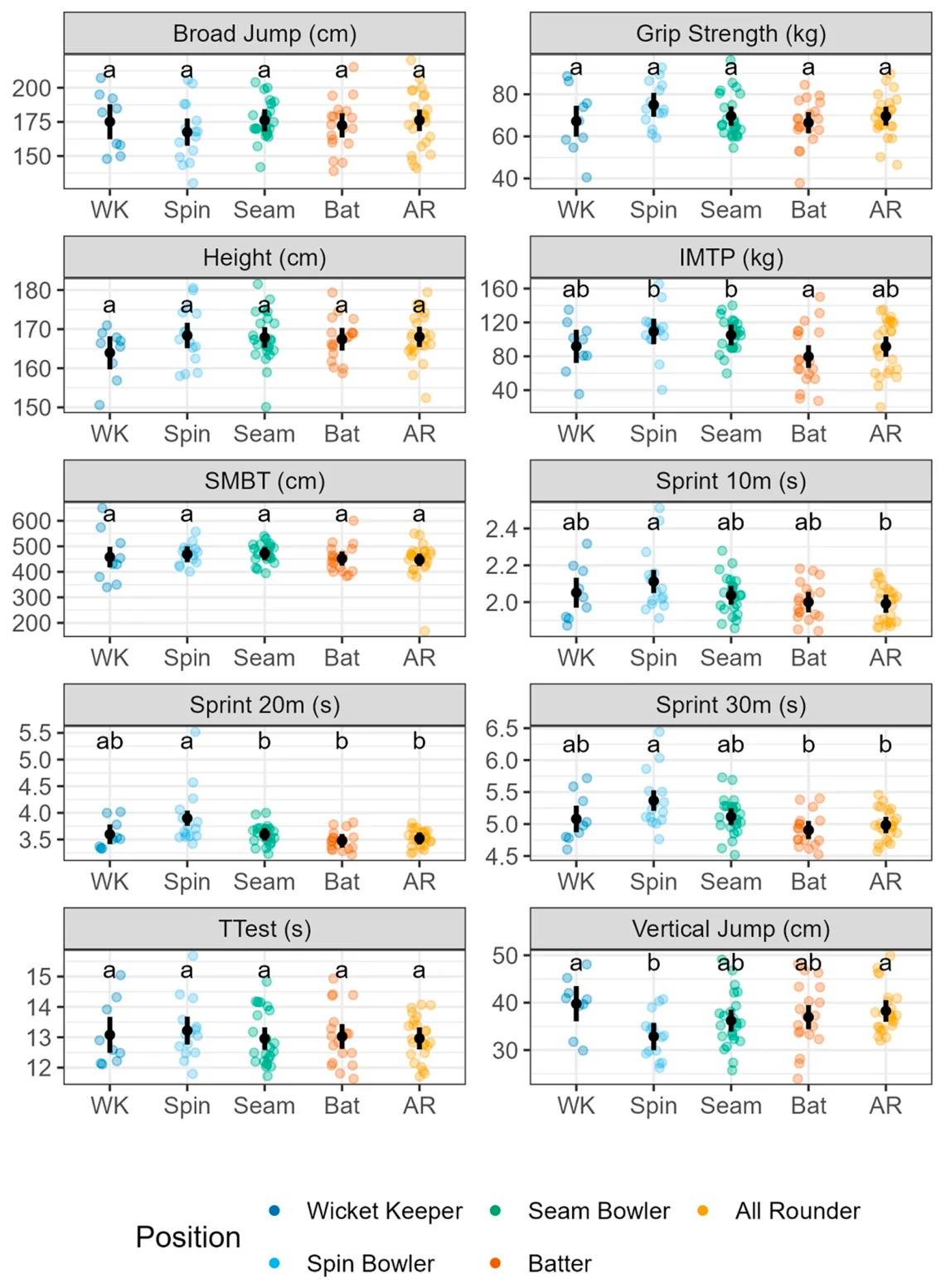
Observed data (coloured dots) and estimated means and 95% confidence intervals (black dots and segments) for physical performance characteristics by position. The compact letter display shows results of post hoc pairwise comparisons. For each outcome, any two groups that do not share a letter are statistically significant from each other (*p* < 0.05). Notes: cm, centimetres; kg, kilograms; s, seconds; AR, all-rounder; Bat, Batter; Seam, Seam bowler; Spin, Spin Bowler; WK, Wicket Keeper.

Significant differences between playing positions were observed in selected physical performance measures, while others remained consistent across roles as shown in Table 1 and Table 2 and Figure 2.

### 3.4. Power and Strength

Lower-body power, measured via vertical jump, differed significantly between positions (F (4,85) = 2.96, *p* = 0.024), as shown in Table 1 and Table 2. Post hoc comparisons, as shown in Figure 2, indicated that spin bowlers demonstrated lower vertical jump performance compared to all-rounders and wicketkeepers, with batters and seam bowlers showing intermediate values. In contrast, horizontal power assessed by the broad jump did not differ between groups (*p* = 0.662). Maximal strength (IMTP) also differed significantly between positions (F (4,85) = 2.94, *p* = 0.025), with spin bowlers and seam bowlers demonstrating higher strength values compared to batters. Grip strength and upper-body power (SMBT) did not differ significantly between positions.

### 3.5. Speed and Agility

Sprint performance demonstrated clear positional variation. Significant differences were observed at 10 m (F (4,85) = 2.66, *p* = 0.038), 20 m (F (4,85) = 5.71, *p* < 0.001), and 30 m (F (4,85) = 5.24, *p* = 0.001) as shown in Table 1 and Table 2. Spin bowlers consistently recorded slower sprint times compared to all-rounders and batters, particularly over longer distances (20 m and 30 m), while seam bowlers and wicketkeepers demonstrated intermediate performance. No significant differences were observed in agility performance assessed via the *T*-test (F (4,85) = 0.25, *p* = 0.908), indicating similar change-of-direction ability across positions.

### 3.6. Relative Age Effect

**Figure 3:**
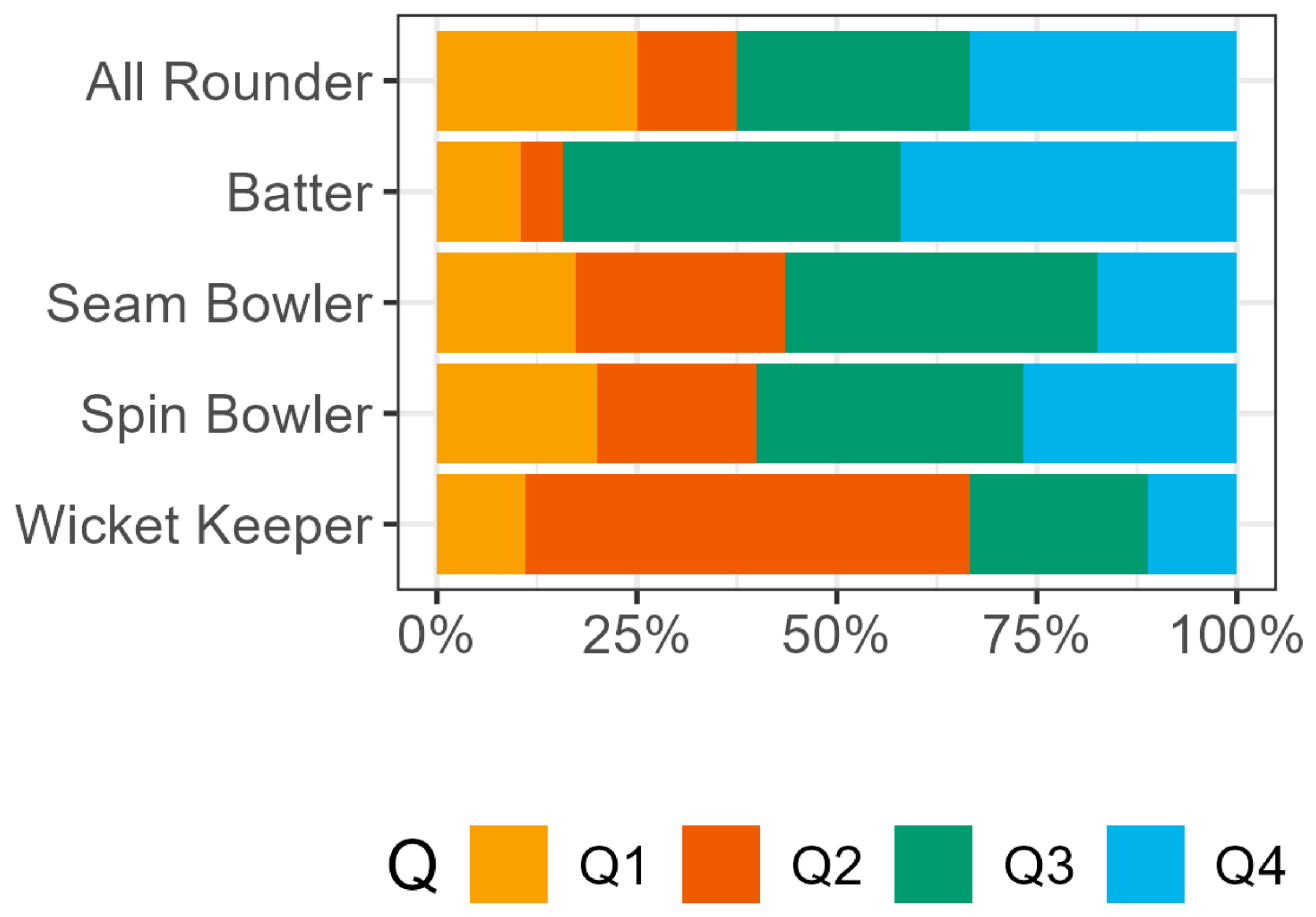
Proportional distribution of players across birth quartiles (Q1–Q4) by playing position.

The distribution of birth quartiles across the cohort showed a relatively balanced pattern, with 18% of players born in Q1, 20% in Q2, 34% in Q3, and 28% in Q4. No significant deviation from a uniform birth quartile distribution was observed (χ^2^ (3) = 6.27, *p* = 0.099, Cramér’s V = 0.26). When examined by playing position, some variability in quartile distribution was evident, as shown in Figure 3. Wicketkeepers were more frequently born in Q2, while batters were more frequently represented in Q3 and Q4. However, these differences were not statistically significant (χ^2^ (12) = 14.92, *p* = 0.246, Cramér’s V = 0.29), and no consistent positional pattern was identified across quartiles.

### 3.7. Maturity Offset Applicability

The modified Moore equation generally produced higher offset values (3.8 ± 1.1 years) and lower estimated age at peak height velocity (APHV; 12.5 ± 0.6 years) compared to the original Mirwald equation (3.4 ± 0.8 years; APHV: 12.9 ± 0.7 years), as shown in Table 1. Both methods indicated that players were well beyond peak height velocity. However, given the mean age of the cohort (16.3 ± 1.3 years), these estimates fall outside the optimal age range for which maturity offset equations are considered most valid (approximately 9–13 years in females). The predicted APHV values were slightly higher than expected norms for female populations (~11–12 years) [20], suggesting a tendency to overestimate maturity timing in this sample. Consistent with previous validation studies [20,22,33], reduced variability and age dependence of predictions were evident, indicating limited sensitivity of maturity offset methods in older adolescent cohorts [11].

## 4. Discussion

The primary aim of this study was to characterise the anthropometric, physical, and developmental profile of elite U19 female cricketers and examine differences across playing roles, relative age distributions, and maturity status. The main findings indicate that (1) physical performance characteristics vary meaningfully across playing positions, particularly in strength, power, and sprint speed; (2) anthropometric differences between roles are minimal; (3) no clear relative age effect is evident in this cohort; and (4) maturity offset equations demonstrate limited sensitivity in this population.

### 4.1. Positional Physical Profiles: Implications for Training

The present findings highlight clear role-specific physical differences, particularly in lower-body power, maximal strength, and sprint performance. Spin bowlers demonstrated lower vertical jump performance and slower sprint times compared to all-rounders and batters, while seam bowlers exhibited higher maximal strength values, as shown in Table 1 and Table 2. These findings align with the biomechanical and physiological demands of each role. Fast bowling requires repeated high-force production and eccentric loading, which is consistent with the higher IMTP values observed in seam bowlers [34]. Conversely, batting and all-round roles involve frequent sprinting, accelerations, and reactive movements, which may explain their superior sprint and power profiles [8,11].

From an applied perspective, these results reinforce the importance of position-specific physical preparation. Previous research highlights that lower-body power is intricately linked to sprint acceleration and change-of-direction ability, which underpin performance in intermittent team sports [10]. Therefore, strength coaches should prioritise explosive power and acceleration-based training in batters and all-rounders, while ensuring that bowlers, particularly spin bowlers, develop sufficient speed capacities alongside their strength base.

For seam bowlers, maintaining high levels of maximal strength and robustness is critical given the repetitive loading associated with bowling actions [8,10,35]. However, the slower sprint performance observed suggests that complementary speed training may be underemphasised in this subgroup. Integrating sprint mechanics and acceleration development may enhance overall athleticism without compromising bowling-specific qualities.

### 4.2. Homogeneity in Anthropometry

Despite differences in physical performance, anthropometric characteristics were, on average, largely similar across positions. This contrasts with findings from male cohorts, where positional differences in height and body mass are more pronounced [8,13,25,34]. The relative homogeneity observed in this female cohort may reflect both selection constraints in developing pathways and lower variation in physical maturation at these ages [13,24]. For practitioners, this suggests that performance differentiation in female cricket is driven more by neuromuscular and physical qualities than by body size alone, reinforcing the importance of targeted training interventions rather than reliance on anthropometric profiling for talent identification.

### 4.3. Relative Age Effect: Limited Influence in Female Cohort

The distribution of birth quartiles did not demonstrate a significant relative age effect (RAE), with a slight skew towards later quartiles (Q3 and Q4). This contrasts with findings in male cricket pathways [25,26], where RAE are typically pronounced and favour relatively older athletes. However, the lack of statistically significant findings in this case may be due to the relatively small sample size (n = 90) and thus insufficient statistical power. Larger studies may be required to determine whether RAE exists among female adolescent cricketers, but inconsistent RAE patterns have been reported in female sport, with studies demonstrating weak or absent effects [36,37]. The absence of a clear RAE in this cohort may reflect the ongoing expansion of women’s cricket and less-established selection pressures compared to male pathways. Additionally, smaller participation pools and differing developmental trajectories have been suggested as contributing factors to reduced RAE prevalence in female populations [38]. From an applied perspective, these findings suggest that player selection within this cohort may be less influenced by chronological age relative to peers. However, continued monitoring is warranted as participation increases and competitive structures evolve.

### 4.4. Maturity Offset: Limited Utility in Late-Adolescent Female Athletes

A key finding of this study is the limited applicability of maturity offset estimation in this population. Both the original Mirwald and modified Moore equations classified players as being well beyond peak height velocity, with minimal variability between individuals, as shown in Table 1 and Table 2. Additionally, the modified equation tended to produce slightly earlier estimates of APHV compared to the original. These findings are consistent with validation studies demonstrating that maturity offset equations are most accurate around the period of peak height velocity and lose sensitivity in older adolescents [20,39]. As chronological age increases, predicted maturity offset becomes increasingly dependent on age itself, reducing its ability to discriminate between individuals. Furthermore, evidence suggests that these equations may systematically misestimate timing of maturation, particularly in homogeneous or elite populations [40,41].

In the present cohort, estimated APHV values (~12.5–12.9 years) were slightly higher than expected norms for female populations (typically ~11–12 years), suggesting a tendency to overestimate maturity timing [19]. This is particularly relevant in applied settings, where overreliance on maturity offset may misinform training or selection decisions. For strength coaches, these findings indicate that maturity offset should not be used in isolation for athlete monitoring or grouping in late-adolescent female athletes. Instead, practitioners should prioritise direct performance metrics (strength, power, speed), longitudinal tracking of individual development, and practical maturity indicators.

### 4.5. Limitations and Future Directions

Several limitations should be considered when interpreting the findings of this study.

First, the cross-sectional design limits the ability to infer causal relationships or examine the longitudinal development of physical and maturational characteristics. Given the known variability in adolescent growth and performance trajectories, future longitudinal studies are warranted to better understand how physical qualities and maturation interact over time in female cricket pathways.

Second, although the sample represents elite U19 female cricketers competing at the highest national school level, the overall sample size, particularly within positional subgroups, was relatively small. This may have limited the statistical power to detect subtle differences between positions and reduced the robustness of subgroup analyses, particularly for relative age effect (RAE) comparisons.

Third, the assessment of biological maturity relied on non-invasive maturity offset equations. As highlighted in the results, these methods demonstrated limited sensitivity in this older adolescent cohort and may have overestimated maturity timing. Previous research has shown that the validity of maturity offset decreases with increasing age and distance from peak height velocity, particularly in female populations. Future research should consider incorporating alternative or complementary markers of maturation, such as longitudinal growth tracking or, where feasible, more precise biological indicators.

Fourth, the study focused primarily on physical performance characteristics and did not include technical, tactical, or psychological measures, which are also critical determinants of cricket performance. Integrating multidimensional performance metrics would provide a more comprehensive understanding of talent and development in female cricket.

## 5. Conclusions

This study provides a comprehensive profile of anthropometric, physical, and developmental characteristics in elite U19 female cricketers. Differences between playing positions were evident in strength, power, and sprint performance, while anthropometric characteristics and agility were largely similar across roles. No clear relative age effect was observed, and maturity offset methods demonstrated limited applicability in this late-adolescent cohort. Collectively, these findings suggest that physical performance qualities, rather than anthropometry or maturity status, are the primary determinants distinguishing players at this level.

### Practical Applications

The findings of this study provide several important implications for strength coaches and cricket practitioners working within elite female youth pathways:

**1. Implement position-specific physical development**

Batters and all-rounders: prioritise sprint acceleration, lower-body power, and repeated high-intensity efforts. Seam bowlers: focus on maximal strength development and load tolerance, while integrating speed training. Spin bowlers: target improvements in power and sprint performance to enhance overall athletic capacity.

**2. Prioritise neuromuscular qualities over anthropometry**

Limited differences in body size suggest that strength, power, and speed are more important discriminators of performance than anthropometric characteristics. Talent identification should emphasise physical capacity and skill execution rather than body size alone.

**3. Use caution when applying maturity offset**

Maturity offset equations may lack sensitivity in older adolescent female athletes. Practitioners should avoid relying on these methods in isolation and instead focus on longitudinal performance monitoring and individual development trends.

**4. Monitor but do not overemphasise relative age**

The absence of a strong RAE suggests reduced selection bias in this cohort. However, as participation increases, practitioners should continue to monitor potential biases within talent pathways.

**5. Emphasise speed–power development**

Strong relationships between power and sprint performance highlight the importance of plyometric training, sprint mechanics, and strength–power integration.

## Figures and Tables

**Table 1:** Physical performance characteristics and anthropometric profile by playing position.

Characteristic	All-Rounder *N* = 24 ^1^	Batter *N* = 19 ^1^	Seam Bowler *N* = 23 ^1^	Spin Bowler *N* = 15 ^1^	Wicket Keeper *N* = 9 ^1^	Overall *N* = 90 ^1^
Height (cm)	168.0 (6.2)	167.4 (5.3)	167.9 (6.6)	168.4 (7.3)	164.0 (6.6)	167.5 (6.3)
Sitting Height (cm)	89.1 (3.2)	89.5 (2.6)	88.4 (5.6)	89.8 (3.6)	89.4 (4.3)	89.2 (4.0)
Leg Length (cm)	78.9 (4.3)	77.9 (3.9)	79.5 (4.1)	78.5 (4.7)	74.5 (3.1)	78.3 (4.3)
Weight (kg)	63.9 (8.5)	63.0 (8.1)	64.6 (7.7)	72.2 (16.5)	66.3 (12.4)	65.5 (10.6)
Hand Dominance						
Left	2 (8.3%)	2 (11%)	1 (4.3%)	1 (6.7%)	0 (0%)	6 (6.7%)
Right	22 (92%)	17 (89%)	22 (96%)	14 (93%)	9 (100%)	84 (93%)
Vertical Jump (cm)	38.3 (4.8)	36.9 (6.7)	36.2 (5.8)	32.9 (4.7)	39.8 (5.7)	36.7 (5.8)
Broad Jump (cm)	176.2 (21.5)	172.4 (18.7)	176.2 (14.6)	167.5 (21.9)	175.1 (21.7)	173.8 (19.3)
IMTP (kg)	91.5 (32.6)	79.6 (35.3)	105.1 (19.5)	109.3 (28.9)	91.8 (30.6)	95.5 (30.9)
Grip Strength (kg)	69.6 (10.2)	66.5 (11.0)	69.7 (10.5)	75.0 (10.2)	67.2 (15.6)	69.6 (11.2)
SMBT (cm)	446.8 (72.5)	451.8 (54.0)	471.8 (37.0)	468.8 (40.2)	457.9 (104.0)	459.0 (60.2)
*T*-test (s)	13.0 (0.7)	13.0 (1.0)	13.0 (0.9)	13.2 (1.0)	13.1 (1.1)	13.0 (0.9)
Sprint 10 m (s)	2.0 (0.1)	2.0 (0.1)	2.0 (0.1)	2.1 (0.2)	2.1 (0.2)	2.0 (0.1)
Sprint 20 m (s)	3.5 (0.2)	3.5 (0.2)	3.6 (0.2)	3.9 (0.5)	3.6 (0.3)	3.6 (0.3)
Sprint 30 m (s)	5.0 (0.2)	4.9 (0.2)	5.1 (0.3)	5.4 (0.4)	5.1 (0.4)	5.1 (0.3)
Age (yrs)	16.1 (1.4)	16.4 (1.3)	15.8 (1.3)	16.4 (1.1)	17.3 (1.1)	16.3 (1.3)
Mirwald Offset	3.3 (0.9)	3.5 (0.9)	3.1 (0.9)	3.5 (0.6)	3.8 (0.8)	3.4 (0.8)
Moore Offset	3.7 (1.1)	3.9 (1.1)	3.5 (1.1)	4.0 (0.8)	4.3 (0.9)	3.8 (1.1)
Mirwald APHV	12.8 (0.7)	12.9 (0.6)	12.7 (0.8)	12.9 (0.8)	13.5 (0.7)	12.9 (0.7)
Moore APHV	12.4 (0.5)	12.5 (0.4)	12.3 (0.5)	12.5 (0.7)	13.0 (0.6)	12.5 (0.6)
Mirwald formula						
OM	14 (58%)	11 (58%)	15 (65%)	10 (67%)	2 (22%)	52 (58%)
LM	10 (42%)	8 (42%)	8 (35%)	5 (33%)	7 (78%)	38 (42%)
Moore formula						
OM	20 (83%)	16 (84%)	21 (91%)	13 (87%)	6 (67%)	76 (84%)
LM	4 (17%)	3 (16%)	2 (8.7%)	2 (13%)	3 (33%)	14 (16%)
Quartiles						
Quartile 1	6 (25%)	2 (11%)	4 (17%)	3 (20%)	1 (11%)	16 (18%)
Quartile 2	3 (13%)	1 (5.3%)	6 (26%)	3 (20%)	5 (56%)	18 (20%)
Quartile 3	7 (29%)	8 (42%)	9 (39%)	5 (33%)	2 (22%)	31 (34%)
Quartile 4	8 (33%)	8 (42%)	4 (17%)	4 (27%)	1 (11%)	25 (28%)

Notes: cm, centimetres; kg, kilograms; s, seconds; m, metres; yrs, years; APHV, Age at Peak Height Velocity; OM, On-time maturers; LM, Late maturers; ^1^ Mean (SD); n (%); IMTP, isometric mid-thigh pull; SMBT, seated medicine ball throw.

**Table 2:** Analysis of variance results for differences between playing positions.

Characteristic	η2	F (4,85)	*p*-Value
Height (cm)	0.04	0.83	0.5100
Vertical Jump (cm)	0.12	2.96	0.0244 *
Broad Jump (cm)	0.03	0.60	0.6620
IMTP (kg)	0.12	2.94	0.0250 *
Grip Strength (kg)	0.06	1.36	0.2540
SMBT (cm)	0.03	0.66	0.6190
*T*-test (s)	0.01	0.25	0.9080
Sprint 10 m (s)	0.11	2.66	0.0383 *
Sprint 20 m (s)	0.21	5.71	0.0004 *
Sprint 30 m (s)	0.20	5.24	0.0008 *

Notes: cm, centimetres; kg, kilograms; s, seconds; m, metres; IMTP, isometric mid-thigh pull; SMBT, seated medicine ball throw; * statistically significant result (*p* < 0.05).

## Data Availability

The original contributions presented in this study are included in the article. Further inquiries can be directed to the corresponding author.

## Abbreviations

The following abbreviations are used in this manuscript:

OM	On-time maturers
LM	Late maturers
ICC	International Cricket Council
APHV	Age at Peak Height Velocity
IMTP	Isometric Mid-thigh Pull
SMBT	Seated Medicine Ball Throw
RAE	Relative Age Effect
MMO	Modified Maturity Offset
PHV	Peak Height Velocity
AR	All-rounder
Bat	Batter
Seam	Seam Bowler
Spin	Spin Bowler
WK	Wicket Keeper
Q1–Q4	Birth Quartile
